# Habitat-Associated Dietary Plasticity in the Japanese Weasel (*Mustela itatsi*): Fecal Analysis in a Floodplain Wetland and Comparative Synthesis

**DOI:** 10.3390/ani16111720

**Published:** 2026-06-04

**Authors:** Shufan Qiao, Kaoru Suzuki, Masato Yoshikawa, Chris Newman, Yayoi Kaneko

**Affiliations:** 1 Carnivore Ecology and Conservation Research Group, Institute of Agriculture, Tokyo University of Agriculture and Technology, Saiwaicho 3-5-8, Fuchu 183-8509, Tokyo, Japan; s243506u@st.go.tuat.ac.jp (S.Q.); ykaneko@cc.tuat.ac.jp (Y.K.); 2 Field Science Centre, Tokyo University of Agriculture and Technology, Saiwaicho 3-5-8, Fuchu 183-8509, Tokyo, Japan; kaoru@cc.tuat.ac.jp; 3 Institute of Agriculture, Tokyo University of Agriculture and Technology, Saiwaicho 3-5-8, Fuchu 183-8509, Tokyo, Japan; masato@cc.tuat.ac.jp; 4 Department for Continuing Education, University of Oxford, Rewley House, 1 Wellington Square, Oxford OX1 2JA, UK

**Keywords:** Japanese weasel, wetland habitat, scat analysis, dietary composition, trophic plasticity

## Abstract

The range of food resources consumed by opportunistic carnivores is shaped by habitat-specific availability and productivity. However, baseline data on the diet of the Japanese weasel (*Mustela itatsi*) in managed wetland habitats remain scarce. Firstly, we conducted a diet study examining Japanese weasel scats from the Watarase-yusuichi wetland (WYW) (September 2024–August 2025). Based on 103 fecal samples, we calculated seasonal frequency of occurrence (FO) for 16 food categories and estimated their relative volume (RV) using the point-frame method. Surprisingly, plant material and fruit seeds were eaten frequently across seasons, and fruit seeds constituted a major component of scat volume, particularly in summer. Secondly, we compared baseline WYW data with published datasets from two contrasting aquatic habitats (a suburban river area and a rice paddy landscape) using harmonized categories and multivariate analyses. Diet composition differed markedly among study sites, and the PERMANOVA analysis indicated a significant effect of sampling site (R^2^ = 0.643, *p* = 0.001) with a weaker seasonal effect (R^2^ = 0.208, *p* = 0.038), with no significant pairwise differences between seasons (all *p* ≥ 0.300). Although cross-site comparisons should be interpreted cautiously because datasets were collected in different decades (1998, 2018, and 2024–2025), these findings support habitat-associated trophic plasticity in the Japanese weasel, with dietary shifts across habitats and seasons apparently reflecting variation in available food resources. In managed floodplain wetlands such as WYW, maintaining a mosaic of reed beds, ponds, and channels may help sustain dietary flexibility and population persistence in this species.

## Simple Summary

Trophic plasticity allows small- and medium-sized carnivores to adapt to anthropogenic habitats by shifting diet in response to local resource conditions. Japanese weasels are flexible hunters, but little is known about their diet adaptation to wetland habitats. This matters in actively managed wetlands, where understanding which foods local predators rely on can inform conservation decisions. We studied Japanese weasel diet over a full year in the Watarase-yusuichi wetland in Japan, using 103 dropping (scats) collected between September 2024 and August 2025. Surprisingly, plant material and fruit seeds were eaten frequently across seasons, and fruit seeds were often a major scat component. Diet also varied strongly among sampling site locations, while seasonal differences were weaker. When contrasted with published information from two other nearby human-modified aquatic habitats (a suburban river area and a rice paddy landscape), we found clear habitat-specific differences. Overall, our study reinforces knowledge of how Japanese weasels adjust their diet across different habitats and seasons, presumably in response to variation in available food resources. These baseline results can support future monitoring and management of wetland habitats and their wildlife.

## 1. Introduction

Many small- and medium-sized carnivores (including many mustelids [1]), particularly those with flexible, generalist diets, have proven able to persist in highly human-modified landscapes. These species can exploit novel foraging opportunities and find suitable shelter within managed green spaces without being overly conspicuous or causing unacceptable disturbance [2]. In urban and periurban settings, these opportunistic carnivores often perform dietary switching [3] according to the local availability of prey and seasonal fluctuations, including taking food associated with human activities [2,4,5,6]. This dietary flexibility provides a key mechanism enabling population persistence under rapid land-use change, benefitting from anthropogenic resource subsidies [7,8,9]. At larger scales, trophic plasticity is shaped by two interacting drivers: habitat structure (determining prey communities, encounter rates, predation risk, and access to aquatic versus terrestrial resources [10,11]) and seasonality (resource pulses and phenological shifts that alter which foods are more “cost-effective” or accessible [12]). Consequently, applying a framework combining habitat and season is central to understanding how small- to -medium- sized carnivores exploit heterogeneous landscapes [13,14,15].

The Japanese weasel (*Mustela itatsi*) is endemic to the main islands of Honshu, Shikoku, and Kyushu in the Japanese archipelago [16]. Due to intra-guild competitive pressures from the invasive Siberian weasel (*M. sibirica*) and habitat modification in lowland areas, Japanese weasel populations are in decline, causing it to be listed as a species of conservation concern on National and Provincial Red Lists [17], and to be classified as Near Threatened (NT) on the IUCN Red List [18,19,20,21]. To better understand the pressures faced by the Japanese weasel, especially those factors affecting its coexistence with the Siberian weasel [20], it is therefore vital to establish a clearer understanding of its trophic plasticity and dietary breadth across human-modified habitats and seasons. How Japanese weasels exploit important large, artificial floodplain wetlands is of particular interest. Such habitats contain reed marshes, aquatic margins, levees, and adjacent terrestrial vegetation, generating high spatial heterogeneity and potential cross-habitat subsidies. To date, however, studies have been limited to riparian corridors in western Tokyo (e.g., Hamura: suburban river landscapes//Tama river corridor [22]), lowland rice paddy landscapes in Saitama Prefecture (e.g., Kazo//Saitama [23]), and other broader suburban data from Ibaraki Prefecture [24,25,26] in the Kantō region, north of Tokyo. These studies used fecal and/or stomach content analysis to show that the Japanese weasel consume a range of terrestrial and semi-aquatic vertebrates and invertebrates, as well as fruits and other plant material, with diet varying among habitats and seasons [23,24,26,27,28,29].

Year-round diet data are also important to understand Japanese weasel foraging decisions in extensive riverine floodplain wetland habitats. This is because studies have identified their seasonal consumption of fruits/plant material [24,28]. This is unusual compared to other *Mustela* spp., which are typically obligate carnivores with physiological and energy requirements met almost entirely by animal prey, such as small mammals, birds, eggs, insects, reptiles, and amphibians [30]. However, direct comparisons across habitats prove challenging due to differences in seasonal food category classification schemes and quantitative metrics (e.g., FO, volume indices, and dry weight ratios) between studies. These variations can obscure patterns when synthesizing scat-based diet studies [31,32,33,34,35]. Consequently, few studies have explicitly partitioned the relative contributions of habitat and season using multivariate designs [33,36].

To address the need for more complete baseline data on Japanese weasel diet in wetland systems [23,27,28], here we conducted a two-step study design. In our first step, we performed a year-round fecal analysis study at the Watarase-yusuichi wetland (hereafter, WYW)—a 2861 ha reed-managed marsh-type floodplain wetland—where Siberian weasels are not currently recorded [20,37], and dietary competition between Siberian weasels and Japanese weasels is therefore unlikely at the study site. We classified 16 key food categories and quantified dietary composition using frequency of occurrence (FO) and relative volume (RV) (Table 1), and examined seasonal variation within WYW.

In the second step, we used a harmonized cross-site comparison to evaluate habitat-specific dietary patterns differences across seasons between WYW and two contrasting habitats: a suburban river corridor (Hamura) and a rice paddy landscape (Kazo).

We tested the hypotheses that Japanese weasel diet at WYW will:

**H1.**  *Be diverse and show seasonal shifts in dietary composition;*

**H2.**  *(a) Rely on aquatic food resources and (b) include fruits/plant material.*

In addition, in step 2, we then tested whether the Japanese weasel diet we establish for WYW;

**H3.**  *Differs from diets established previously for a suburban riverine habitat (Hamura [27]) and a rice paddy landscape (Kazo [23,38]), reflecting broad opportunism in relation to food resource availability (Table 2). Because the comparison relied on datasets collected in different periods, H3 was treated as an exploratory cross-site comparison.*

We ultimately use our findings to make habitat management and conservation recommendations for Japanese weasels in wetland areas.

**Table 1:** Frequency of occurrence (FO%) (**a**) and relative volume (RV%) (**b**) of Japanese weasel diet in Watarase-yusuichi between September 2024 and August 2025, as revealed through fecal analysis.

a. Frequency of Occurrence (%)																	
	N	Mammals	Birds	Reptiles and Amphibians	Fish	Coleoptera Insects	Orthoptera Insects	Insect Larvae	Other Insects	Crustaceans	Earthworms	Other Animals	Seeds (Fruits)	Leaves	Other Plants	Artificial Objects	Others
Autumn	25	16.0	20.0	0.0	8.0	48.0	8.0	4.0	24.0	0.0	0.0	0.0	28.0	32.0	40.0	0.0	32.0
Winter	36	30.6	16.7	0.0	8.3	0.0	0.0	0.0	8.3	0.0	0.0	0.0	58.3	50.0	80.6	2.8	30.6
Spring	22	31.8	9.1	9.1	36.4	18.2	22.7	4.5	0.0	9.1	0.0	4.5	27.3	18.2	36.4	0.0	18.2
Summer	20	5.0	0.0	0.0	5.0	70.0	0.0	0.0	5.0	0.0	0.0	0.0	80.0	0.0	35.0	0.0	0.0
Total	103	22.3	12.6	1.9	13.6	29.1	6.8	1.9	9.7	1.9	0.0	1.0	48.5	29.1	52.4	1.0	22.3
**b. Relative Volume (%)**																	
	**N**	**Mammals**	**Birds**	**Reptiles and** **Amphibians**	**Fish**	**Coleoptera** **Insects**	**Orthoptera** **Insects**	**Insect** **Larvae**	**Other** **Insects**	**Crustaceans**	**Earthworms**	**Other** **Animals**	**Seeds** **(Fruits)**	**Leaves**	**Other** **Plants**	**Artificial** **Objects**	**Others**
Autumn	25	10.9	5.3	0.0	4.2	30.3	4.3	1.1	11.4	0.0	0.0	0.0	9.8	3.5	18.0	0.0	1.1
Winter	36	19.5	7.6	0.0	7.8	0.0	0.0	0.0	0.1	0.0	0.0	0.0	39.0	14.6	11.0	0.0	0.4
Spring	22	25.8	4.8	4.7	15.4	6.4	10.4	4.2	0.0	5.3	0.0	0.1	8.2	3.3	11.0	0.0	0.6
Summer	20	5.0	0.0	0.0	4.9	29.0	0.0	0.0	0.2	0.0	0.0	0.0	54.1	0.0	7.0	0.0	0.0
Total	103	15.9	5.0	1.0	8.0	14.3	3.3	1.2	2.8	1.1	0.0	0.0	28.3	6.6	11.9	0.0	0.5

**Table 2:** Frequency of occurrence (FO%) of food categories in the diet of Japanese weasels across different sites/habitat types and seasons in the Kanto region, based on previous studies and the current study (N, number of feces). Sites are abbreviated as ‘HAM’, Hamura; ‘KAZ’, Kazo; and ‘WYW’, Watarase-yusuichi Wetland. Seasons are abbreviated as ‘Au’, autumn; ‘Wi’, winter; ‘Sp’, spring; and ‘Su’, summer.

Site	HAM, Tokyo				KAZ, Saitama				WYW, Tochigi			
Habitat type	Riverine, suburban				Paddy fields				Riverine wetland			
Season	Au	Wi	Sp	Su	Au	Wi	Sp	Su	Au	Wi	Sp	Su
N	94	71	55	65	23	21	11	20	25	36	22	20
Food category												
Mammals	18.9	24.3	30.7	12.5	4.3	4.8	18.2	0.0	16.0	30.6	31.8	5.0
Birds	6.7	3.4	2.8	4.3	0.0	0.0	0.0	0.0	20.0	16.7	9.1	0.0
Reptiles and amphibians	1.8	10.3	10.1	0.0	56.5	14.3	72.7	25.0	0.0	0.0	9.1	0.0
Fish	20.3	16.2	19.5	11.0	0.0	9.5	0.0	0.0	8.0	8.3	36.4	5.0
Insects	19.2	1.8	9.9	25.2	52.2	9.5	90.9	35.0	56.0	8.3	36.4	75.0
Crustaceans	24.4	37.8	9.8	30.6	65.2	14.3	36.4	45.0	0.0	0.0	9.1	0.0
Other animals	3.6	0.0	3.9	2.6	26.1	23.8	18.2	35.0	0.0	0.0	4.5	0.0
Seeds (fruits)	1.7	1.6	0.9	0.7	13.0	66.7	9.1	10.0	28.0	58.3	27.3	80.0
Other plants	2.8	3.2	4.6	13.0	43.5	61.9	45.5	15.0	56.0	83.0	45.5	35.0
Reference	Fujii et al. (1998) [27]				Mitsui (2018) [38], Tsunoda (2024) [23]				This study			

## 2. Materials and Methods

### 2.1. Study Area

This study was conducted at Watarase-yusuichi wetland (WYW), an artificially restored and managed 2861-hectare riverine floodplain wetland designated as a National Wildlife Protection Zone and listed as a Ramsar Site (Site No. 2061). WYW spans four prefectures (Ibaraki, Tochigi, Gunma, Saitama) on Honshu Island, Japan (36°14′20″ N, 139°40′56″ E), lying approximately 60 km north of Tokyo. The site sits at the confluence of the Watarase, Uzuma, and Omoi rivers and includes the Watarase Reservoir, which provides an artificial floodwater retarding basin enclosed by embankments, primarily used for flood control management within the Tone River system [39].

Within Japan’s evergreen forest biogeographic region, WYW is representative of a low-moor wetland habitat dominated by common reed (*Phragmites australis*). WYW includes extensive reed vegetation, ponds, and channels. Site information records approximately 1000 plant species, 275 bird species, 1700 insect species, and 78 fish and shellfish species, suggesting that WYW provides potential food resources for Japanese weasels across both aquatic and reed bed habitats [40]. Beyond flood control, WYW is used for recreational activities (including hiking and fishing) and environmental education. Sediment accumulation poses a drying risk, and sediment removal has been conducted periodically since 2010 [39].

**Figure 1:**
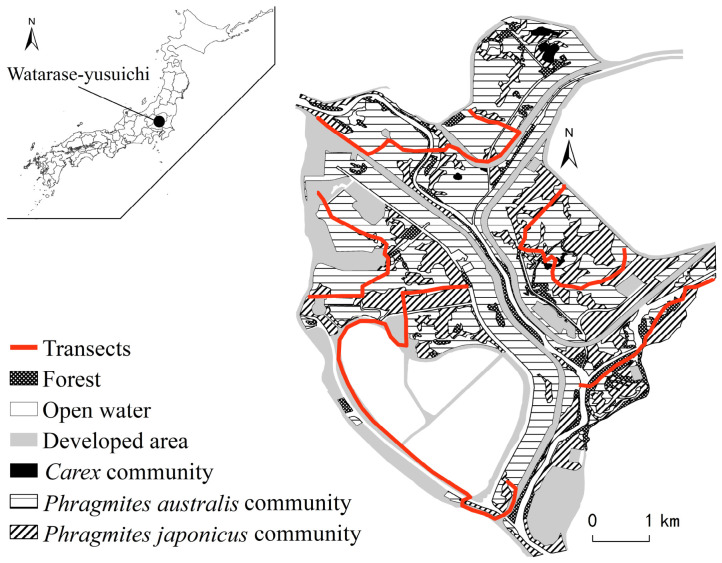
Location and land-cover map of Watarase-yusuichi, Japan. Vegetation communities and other land-cover types are shown based on data sourced from the official Watarase-yusuichi website (Watarase-yusuichi official website; accessed 3 November 2025).

To produce a vegetation type/habitat classification map, we applied the same vegetation categories as defined by the Watarase Floodplain official website [41], see Figure 1. For subsequent analyses, these vegetation types were grouped into broader habitat classes. Regional climate data were obtained from the Japan Meteorological Agency (1991–2020 climate normals dataset; observation point: Koga, Ibaraki Prefecture, ~5 km southeast of Watarase-yusuichi), with an average annual temperature of 14.9 °C and average annual precipitation of 1229.9 mm [42].

### 2.2. Fecal Sample Collection

Japanese weasel fecal samples were collected weekly from September 2024 to August 2025 by walking along five standardized survey transect polylines (total combined distance, 27 km) and surveying a 2 m strip on either side to maximize scat detection probability [43,44,45]. These transects followed accessible embankments, maintenance roads, and trails that traversed all defined habitat categories (Figure 1).

Each scat collection location was recorded using GPS, along with the date. Japanese weasel scats were distinguished from those of other sympatric carnivores at WYW (e.g., raccoon dog *Nyctereutes procyonoides viverrinus* and red foxes *Vulpes vulpes* [46]), based on their smaller volume/diameter (≤10 mm [47,48]) and characteristic appearance in combination with other diagnostic features, including shape and texture, presence of nearby tracks or feeding signs, prey hair within the scat, and characteristic odor [49]. All scat identification was conducted by the first author using these standardized field criteria throughout the study; therefore, no formal inter-observer reliability test was required, and no fecal DNA confirmation was conducted. Therefore, occasional misidentification of scats from sympatric carnivores cannot be completely excluded, although multiple diagnostic features were used together to minimize this risk. Each fecal sample was collected individually, sealed in a plastic bag, transported back to the laboratory, and stored at −20 °C until analysis [23]. Because individual weasels were not identified genetically, each scat was treated as an independent dietary sample; however, repeated contributions by the same individuals across weekly surveys cannot be ruled out. For subsequent analyses, seasons were defined as: spring (March–May), summer (June–August), autumn (September–November), and winter (December–February).

### 2.3. Dietary Analysis

Thawed fecal samples were processed and analyzed according to established procedures [47,50,51]. Each fecal sample was rinsed with 1 L of water through a 1 mm × 1 mm mesh size sieve. Undigested residues retained in the sieve were transferred to a Petri dish and examined under ×30 magnification to identify food items [24]. To detect earthworm chaetae, the rinse water was collected in a glass flask and allowed to settle for ~15 min, then 10–15 mL of bottom sediment was collected with a pipette, plated on a Petri dish, and examined at ×20 magnification [24].

Food items recovered from feces were categorized into: (1) Mammals, (2) Birds, (3) Reptiles and amphibians, (4) Fish, (5) Coleoptera, (6) Orthoptera, (7) Insect larvae, (8) Other insects, (9) Crustaceans, (10) Earthworm, (11) Other animals, (12) Seeds (fruits; implying fruit consumption), (13) Leaves, (14) Other plants (e.g., stems or roots), (15) Artificial objects, (16) Other. Note: “Other insects” and “other animals” denoted unidentifiable fragmented insect/animal residues (e.g., exoskeletal fragments, unrecognizable bone pieces) that could not be assigned to specific taxa [24]. “Artificial objects” included items potentially originating from human activities, such as refuse or agricultural materials [24].

Diet composition was quantified using frequency of occurrence (FO) and relative volume (RV). FO was calculated following Kaneko et al. [24] and Hisano et al. [52]:

$$\mathrm{FO}=\frac{number\;of\;faeces\;in\;which\;the\;food\;category\;occurs}{\mathrm{total}\;\mathrm{number}\;\mathrm{of}\;\mathrm{feces}}\times 100$$

Relative volume (RV) was estimated for each fecal sample using the point-frame method [53]. Washed residues were spread evenly in a Petri dish and quantified by recording item categories at intersections of a gridded frame; the proportion of points assigned to each category was used as an estimate of its proportional volume [54]. RV was calculated as follows:

$$\mathrm{RV}=\frac{number\;of\;food\;category\;ccurrences\;at\;frame\;points}{\mathrm{total}\;\mathrm{number}\;\mathrm{of}\;\mathrm{points}}\times 100$$

FO and RV were interpreted as complementary but different metrics of diet composition. FO indicates how frequently a food category occurred among fecal samples and is useful for detecting commonly used items, but it can overemphasize items consumed frequently in small amounts. RV estimates the proportional volume of identifiable undigested residues within feces and may more closely reflect the relative volumetric contribution of food categories than FO. However, because both indices are based on undigested remains, they are affected by differences in digestibility and detectability among food categories and should not be interpreted as direct measures of ingested biomass or energetic contribution [31,32,33].

### 2.4. Statistical Analyses

Within WYW, we summarized diet composition using FO and RV across seasons and sampling locations. Seasonal variation was assessed descriptively. Because the number of scats differed among seasons (autumn: 25, winter: 36, spring: 22, summer: 20), and no rarefaction or resampling procedure was applied, seasonal FO comparisons, especially for rare food categories, were interpreted cautiously.

We then compared our WYW data with published studies from Hamura (suburban riverine) and Kazo (rice paddy). Both published comparison datasets were based on fecal analysis, so the cross-study comparison used the same general type of dietary evidence. Because these datasets were collected in different periods (HAM: 1998; KAZ: 2018; WYW: 2024–2025), this cross-site comparison should be interpreted as an exploratory comparison of site-associated dietary patterns rather than as a fully replicated test of habitat effects. To facilitate multivariate analysis, we reclassified our data from WYW into consistent, harmonized categories. Cross-study multivariate comparisons were based on FO rather than RV because FO values were consistently available and could be harmonized across WYW, HAM, and KAZ, whereas RV estimates were not available in a directly comparable form among the published datasets. Consequently, some FO values for WYW differ between Table 1 and Table 2. Since Fujii et al. [27] did not report the targeted detection for earthworms, where a lack of detection does not equate to absence, this taxonomic group was excluded from cross-study comparisons. We conducted multivariate analysis using a matrix of FO values across all study site × season combinations (3 sites × 4 seasons = 12 rows; with food categories as columns). Because this matrix contained only 12 site–season combinations, permutation-based tests, particularly pairwise seasonal comparisons, had limited statistical power and were interpreted cautiously. Next, we performed a principal component analysis (PCA) to visualize dietary patterns among different site–season groups. To explore the relative contributions of study site (WYW, HAM, KAZ) and season across studies, we calculated Bray–Curtis dissimilarity [55] from the FO matrix. These dissimilarities were analyzed using a two-factor PERMANOVA (999 permutations) implemented in the R package vegan (version 2.7.2) [36,56]. Homogeneity of multivariate dispersion was also tested using betadisper in vegan, based on Bray–Curtis dissimilarities, followed by permutation tests (permutest, 999 permutations) for both site and season groups. Finally, pairwise comparisons among seasons were performed using permutation-based methods. All analyses were conducted in R (version 4.5.2) [57].

## 3. Results

### 3.1. Diet Composition in the Watarase-Yusuichi Wetland (WYW)

The first step of our study established primary data for WYW. Here, we analyzed a total of 103 fecal samples (n = 25 in autumn, n = 36 in winter, n = 22 in spring, n = 20 in summer; Table 1). The most frequently occurring food category consumed across all seasons was Other plants (FO = 52.4%), followed by Seeds (fruits) (48.5%). Leaves and Coleoptera each had an FO of 29.1% (Table 1a). Insects were also detected frequently, particularly Coleoptera (overall FO = 29.1%), which were the most frequent food category consumed in the summer (FO = 70.0%; Table 1a). Earthworm chaetae were not detected.

Estimates of relative volume (RV) further corroborated the importance of fruits, with the seed category contributing the greatest annual RV (28.3%), peaking in summer (RV = 54.1%) and remaining elevated in winter (RV = 39.0%) (Table 1b). The volumetric contribution of animal prey categories varied substantially with season: mammals and fish contributed more to total RV in the spring than in summer (spring RV: mammals = 25.8%, fish = 15.4%; summer RV: mammals = 5.0%, fish = 4.9%) (Table 1b). Collectively, these results indicate that Japanese weasels at WYW were highly omnivorous throughout the year and that the utilization of plant material was consistently high, while the volumetric contribution of specific prey categories underwent significant seasonal variation.

### 3.2. Cross-Site Comparisons of FO and Seasonal Patterns

In our second step, to place our empirical WYW baseline in a broader context, we compared diet composition with published datasets using harmonized food categories (Table 2). Diet composition differed markedly among the three sites. Japanese weasel diet at WYW showed consistently higher FO for plant-related categories than Hamura (hereafter, HAM) and Kazo (hereafter, KAZ), particularly “Other plants” (WYW FO: autumn 56.0%; winter 83.0%; spring 45.5%; summer 35.0%) and “Seeds (fruits)” (28.0%, 58.3%, 27.3%, 80.0%, respectively) (Table 2). In contrast, KAZ had the highest FO of “Reptiles and amphibians” (autumn FO = 56.5%; spring 72.7%) and “Insects” (autumn FO = 52.2%; spring 90.9%). Japanese weasel diet at HAM included “Fish” consistently across seasons (FO 11.0–20.3%), whereas “Fish” were only recorded in winter at KAZ (FO = 9.5%) and peaked in spring at WYW (FO = 36.4%). Japanese weasel diet at HAM and KAZ also had a higher FO for “Crustaceans” (HAM FO = 9.8–37.8%; KAZ FO = 14.3–65.2%) than at WYW, where “Crustaceans” were consumed only in spring (FO = 9.1%) (Table 2). These comparisons are based on aggregated site × season frequency data from each study.

**Figure 2:**
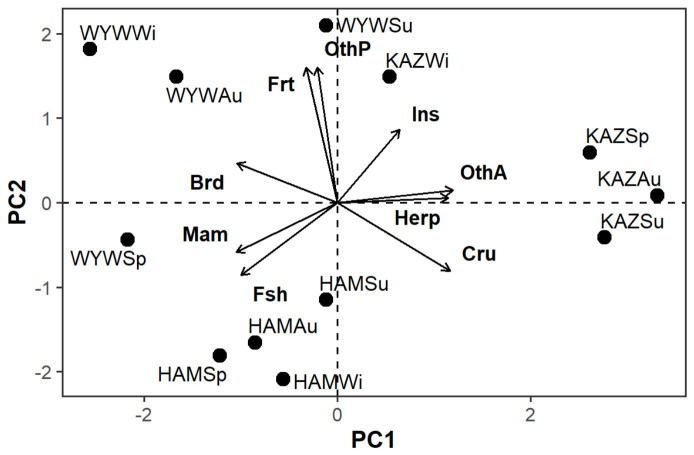
Biplot of food categories and study site–season groups based on the first two principal components (horizontal axis = PC1, vertical axis = PC2). Greater dietary representation of each food category is indicated by arrows. Black circles represent the centroids of site–season combinations (HAM = Hamura, WYW = Watarase-yusuichi Wetland, KAZ = Kazo; Sp = spring, Su = summer, Au = autumn, Wi = winter). The eigenvalues (λ values) for PC1 and PC2 are 42.8% and 24.8%, respectively, explaining a total of 67.6% of the variation in diet composition.

Principal component analysis (PCA) based on FO across 12 ‘site × season’ groups was applied to this comparative dataset (Figure 2). The first two principal components explained 67.6% of the total variance (PC1 = 42.8%, PC2 = 24.8%). PC1 represented animal prey categories, where “Reptiles and amphibians” (0.410), “Crustaceans” (0.417), and “Other animals” (0.429) had positive loadings, while “Mammals” (−0.372), “Birds” (−0.370), and “Fish” (−0.355) had negative loadings (Table 3). PC2 was strongly and positively associated with plant-related categories, particularly “Seeds (fruits)” and “Other plants” (both loading 0.572; Table 3). Consistent with the high representation of plant-related categories, site–season centroids for Japanese weasel diet composition at WYW were positioned higher in the PC2 region of the ordination space, while KAZ centroids were clustered predominantly in the high PC1 region, reflecting a greater contribution from animal prey categories, whereas HAM centroids were in the lower PC2 region (Figure 2).

### 3.3. Effects of Season and Study Site (PERMANOVA)

Within this aggregated cross-site dataset, pairwise comparisons based on Bray–Curtis dissimilarity of FO composition detected no overall significant seasonal differences in Japanese weasel diet among any of the three sites (all *p* ≥ 0.300; Table 4). We then used a two-factor PERMANOVA on the aggregated site × season dataset to assess the relative contributions of study site and season to diet composition. This analysis indicated a significant and substantial effect of study site on diet composition (R^2^ = 0.643, F = 13.023, *p* = 0.001), while season explained a smaller but still significant proportion of variation (R^2^ = 0.208, F = 2.810, *p* = 0.038; Table 5). Multivariate dispersion did not differ significantly among sites (PERMDISP: F = 0.1113, *p* = 0.888) or among seasons (PERMDISP: F = 0.0727, *p* = 0.963).

## 4. Discussion

### 4.1. Step-1: Diet Composition of Japanese Weasels at WYW

Consistent with our first hypotheses (H1), this year-round baseline study of Japanese weasel diet from the Watarase-yusuichi floodplain wetland revealed substantial seasonal opportunistic variations in the FO and RV of the diverse food categories consumed (Table 1). Spring diet was heavily biased toward reptiles and amphibians, fish, orthoptera, and crustaceans, with a strong representation of mammals, birds, seeds (fruits), leaves and other plant matter, consistent with seasonal patterns of food availability in the environment. These seasonal changes were evident not only in FO but also in the relative volumetric contribution of undigested remains in scats (RV; Table 1b). By summer, the diet shifted to be almost entirely dominated by coleoptera and seeds (fruits), along with other plant material and minor FOs of mammals, fish, and other insects. Notably, both the FO and RV of seeds (fruits) were high in summer (FO = 80.0%; RV = 54.1%), showing that fruit comprised a large proportion of the summer diet. In autumn, the FOs of coleoptera and other insects became predominant, along with a modest contribution from mammals, birds, other insects, seeds (fruits), leaves, and other plant material. Substantial dietary shifts then occurred in winter, with high FOs of fruits, leaves, and other plant materials dominating, along with smaller representations of mammals, birds and fish. Seeds (fruits) and leaves also comprised a substantial RV contribution in winter (seeds RV = 39.0%; leaves RV = 14.6%) (Table 1b).

Partially consistent with the first posit of our second hypothesis (H2a), the diet of Japanese weasels at WYW was thus strongly biased toward an intake of aquatic food categories (fish, crustacea, and aquatic coleoptera/orthoptera) in spring. However, the importance of aquatic food categories decreased in other seasons, as diet became dominated by fruits and coleoptera in summer before transitioning to a greater reliance on vertebrate prey through autumn and winter. In spring, this aquatic prey bias was also reflected in RV (e.g., fish RV = 15.4% and crustaceans RV = 5.3%), whereas aquatic categories contributed little to RV in other seasons (Table 1b).

The absence of earthworms in these Japanese weasel feces was also informative. Given that targeted methods were used to detect chaetae, their non-detection suggests that earthworms, if consumed at all, contributed little to the diet at this site. This contrasts with reports from other Japanese landscapes (e.g., rural areas and lowland agricultural landscapes), where earthworms have been recorded to contribute substantially to Japanese weasel diet [23,26,28], suggesting that wetland habitat and substrate conditions at Watarase-yusuichi may limit earthworm availability or accessibility.

Importantly, we found strong support for the second posit of hypothesis two (H2b), where Japanese weasels at WYW did indeed rely strongly on fruits, leaves, and other plant material throughout the year. This summer peak in fruit (seed) consumption (FO = 80%) was consistent with peak environmental availability of fruits. This pattern was reinforced by RV%, with seed volume peaking in summer (RV = 54.1%) and remaining high in winter (RV = 39.0%; Table 1b). Fruits have evolved to be rich in sugars to attract animal seed-disperser mutualists [58], which mustelids can easily digest [59]. Japanese weasels have been established to act as endozoochorous seed dispersers, suggesting a direct functional benefit from their feeding habits at both local and landscape levels [29]. The high frequency of fruit consumption by Japanese weasels in WYW exemplifies their trophic plasticity. Among related mustelids, switching to seasonal fruits is rare among *Mustela* species [60,61,62], although it has been documented [63,64]; this trait is more typically seen in martens (*Martes* spp. [52,65,66,67]). Comparison with the similarly sized Siberian weasel (*Mustela sibirica*) is also relevant because of potential dietary competition between the two species in Japan. Compared with Japanese weasels and *Martes* spp., Siberian weasels are generally described as more carnivorous, with small mammals as important prey items, although plant-derived items have also been recorded in their diet [68,69]. Leaves and the other plant material category were also consistently represented throughout the year, even when other food categories were abundant at WYW, although their occurrence peaked in winter. The lack of leaves in their summer diet—when leaves are abundant—may suggest that they switch away from leaves when more nutritious food sources are available, turning to leaves mostly during autumn and winter.

This consistent occurrence of ‘other plant material’ warrants careful consideration because mustelids, in common with all other Carnivora, except bears (Ursidae—through weak microbial digestion), have a limited capacity to digest cellulose [70,71,72]. The only other report of substantial non-fruit plant consumption by weasels relates to eating fungi [73]. In contrast to fruits, the annual mean FO value (52.4%) for ‘other plant material’ at WYW was substantially higher than the RV value (11.9%, including within individual seasons) (Table 1). This suggests that, while other plant material was consumed frequently, it comprised a relatively small proportion of total fecal volume. Although precise identification of fecal contents can be challenging [32], it was unlikely that we misclassified plant material at these levels of representation. Carnivora species often consume small quantities of plant fiber to facilitate gut motility, expel indigestible material (e.g., hair and bone fragments), and/or reduce parasite burdens [74,75]. Another plausible explanation, warranting future investigation, is that Japanese weasels were actually targeting aquatic larvae of the large reed beetle (*Donacia clavipes*), which occurs commonly among the roots of sedges (*Carex* spp.) and common reeds (*Phragmites* spp.) in WYW’s wetland habitats [76,77]. Therefore, roots of these plants may have been consumed incidentally in small quantities. Supporting this, coleoptera, the taxon and food category to which these large reed beetles belong, were well represented in the overall diet of Japanese weasels at WYW (FO = 29.1%, RV = 14.3%, although not eaten in winter; Table 1). Alternatively, given that phragmites rhizomes store substantial non-structural carbohydrates, including starch and soluble sugars, small quantities of reed rhizomes may have been consumed intentionally, especially in late winter/early spring [78] when other food categories were scarce. Certainly, winter-biased consumption of other plant material, subsidized by fruits and leaves, could be important because Japanese weasels are too small and slender to hibernate [1,79,80]. Whatever the reason for plant material consumption, the indigestibility of any plant vegetative material consumed by weasels [81] would ultimately lead to it being overrepresented in fecal samples.

Mechanistically, the representation of aquatic food categories we detected at WYW (although lower than at KAZ or HAM; see Section 4.2) aligns with the concept that landscape heterogeneity enhances cross-habitat resource availability through spatial subsidies, such that resource inputs generated in aquatic habitats supported Japanese weasels in terrestrial habitats [82]. Thus, the interplay between aquatic/wetland and terrestrial habitats may reshape consumer dietary patterns during seasonal pulses or shifts in resource availability [83,84,85]. In a managed floodplain habitat, such as WYW, where reed beds, levees, roads/pathways, and adjacent terrestrial patches are contiguously juxtaposed, it appears that Japanese weasels can switch effectively between exploitation of food types available in both terrestrial and aquatic habitats [83,85].

### 4.2. Step-2: Comparison with Other Study Sites

Consistent with our third hypothesis (H3), weasel diet at WYW did indeed differ from comparative study sites. However, contrary to the expectation of H2a, Japanese weasel diet at WYW included a lower FO of aquatic resources than reported for the other wetland study sites we compared (Table 2). The seasonal FO values for the crustacean food category in our study were substantially lower than those reported by Fujii et al. [27] for an urban riverine site (HAM) and by Mitsui et al. [38] and Tsunoda et al. [23] for paddy field sites (KAZ). However, we noted a much greater consumption of fish than reported for the paddy field sites, especially in spring, approaching the FO values for fish reported by Fuji et al. [27]. For insects, it was not possible to retrospectively establish which individuals were aquatic larvae and which were adults, either from comparative studies or from fecal analyses conducted at WYW.

These study site contrasts were captured clearly by the multivariate analyses. The PCA separated Watarase-yusuichi along a plant-related axis, while Japanese weasel diet at Kazo clustered along an axis associated with animal prey, especially reptiles and amphibians and invertebrates. Hamura occupied an intermediate position characterized by aquatic prey. PC1 captured variation among animal prey categories (Table 3), aiding in distinguishing components between rice paddy landscapes (i.e., KAZ) and riverine corridors/wetlands (Figure 2). PC2 was loaded by “seeds (fruits)” and “other plants” (Table 3), separating WYW “location × season” groups from other sites along the positive trajectory of PC2 in ordination space (Figure 2).

Since food categories were harmonized for cross-site comparisons (Table 2), some FO values for WYW differ from those calculated using the more detailed classification primarily applied in this study (Table 1). Such discrepancies are expected when items are reclassified into broader categories for comparability. Temporal heterogeneity is another important limitation of this cross-site comparison. The comparison combines datasets collected during different periods (HAM: 1998; KAZ: 2018; WYW: 2024–2025), and dietary patterns at each site may have changed over time. In human-modified landscapes, land-use change and anthropogenic resource subsidies can affect food availability and carnivore diets [7,8,9]; therefor, some observed differences may reflect temporal change and habitat-associated variation. The prominence of reptiles and amphibians and insects at the rice field site (KAZ) likely reflects the rice agricultural ecosystem and its irrigation networks, which form seasonally flooded wetland mosaics that support amphibian breeding and provide abundant invertebrate resources during spring and summer [86,87,88]. Consistent with this ecological context, the frequency of occurrence for reptiles and amphibians at KAZ in spring (FO = 72.7%) was far higher than at WYW (9.1%) and HAM (10.1%) during the same season (Table 2). Similarly, insects comprised a more substantial percentage of the spring diet at KAZ (FO = 90.9%; Table 2). This pattern aligns with the high productivity of flooded rice fields and field margins during the growing season and reflects the Japanese weasel’s tendency to exploit opportunistic prey when encounters are frequent [23].

In the PCA, Japanese weasel diet at KAZ corresponded with the animal–prey structure reflected by PC1 (positively correlated with reptile/amphibian and partially invertebrate-related categories; Table 3), while remaining distinctly separated from the plant-related positioning of WYW along PC2 (Figure 2). In contrast, the diet pattern reported for HAM indicates that Japanese weasels had regular access to aquatic resources along riverbanks in suburban riparian corridors, with fish and crustaceans occurring relatively frequently across seasons [27]. For example, at HAM, fish FO was consistently between 11 and 20% across seasons (e.g., autumn FO = 20.3%), and crustaceans were also relatively common (e.g., winter FO = 37.8%; Table 2). This contrasts with KAZ, where fish were largely absent in most seasons (FO = 0.0 in autumn, spring, and summer; Table 2), and differs from WYW, where fewer crustaceans were detected in Japanese weasel diet only in spring (FO = 9.1%; Table 2). These differences align with habitat-specific prey encounter rate, where continuous flowing water environments may enhance the predictability of aquatic prey availability, whereas in rice paddy mosaics and reed-dominated wetlands, aquatic prey availability is likely to be less reliable. Overall, these results suggest that even within regions with similar regional climatic background conditions, habitat differences may help explain distinct dietary patterns in Japanese weasels [23,27]. The PERMANOVA results reinforce this interpretation: study site (habitat) explained more than three times the variation in diet composition than season. Thus, although seasonal changes occurred within each study site, these are secondary to the overarching influence of local prey availability and encounter rates.

### 4.3. Interpreting Seasonal Effects

Although seasonal variation was evident descriptively at WYW (Step 1), the multivariate analysis across sites (Step 2) indicated that habitat explained substantially more variation than season. Thus, while Japanese weasels adjust resource use within WYW across seasons, broader habitat context exerts stronger influence on overall trophic structure. When seasonal effects were combined across study sites, pairwise comparisons between seasons were not significant (Table 4). However, when both study site (habitat) and season were tested simultaneously (Table 5), season explained a relatively small but statistically significant portion of the variation (R^2^ = 0.208, *p* = 0.038), although the location effect dominated (R^2^ = 0.643, *p* = 0.001). This does not imply an absence of seasonal adjustment; rather, it indicates that seasonal responses are habitat dependent. Within-site seasonal shifts occur, but they differ in direction and magnitude across habitat regions. Ultimately, however, our data were limited to “location × season” combinations as analysis units (12 rows in total), which inherently limited repetition and statistical power, making pairwise seasonal differences harder to detect (Table 4). Nonetheless, the consistent strength of the site effect supports the interpretation that habitat context is the dominant driver of resource use in this system.

### 4.4. Limitations and Future Directions

Fecal analysis based on undigested residues can be affected by biases arising from differences in the digestibility and detectability of prey categories. Soft-bodied prey, such as earthworms, and highly digestible tissues often leave few identifiable traces, while hard structures such as hair, bones, insect exoskeletons, and seeds may be overrepresented. Thus, FO and RV serve better as relative and comparative indicators than as direct measures of actual biomass intake [31,32,33]. Furthermore, the resolution of taxonomic identification and how items are grouped can influence inferred patterns; therefore, subtle category-level differences between sites should not be over-interpreted [32,33]. Cross-site comparisons were also constrained by temporal heterogeneity among datasets (HAM: 1998; KAZ: 2018; WYW: 2024–2025). Thus, site-level dietary differences may partly reflect temporal change as well as habitat-associated variation. In addition, identification of species producing fecal samples was based on morphology, size, field signs, and odor rather than genetic confirmation; therefore, occasional misidentification with sympatric carnivores cannot be entirely excluded. Similarly, because fecal DNA genotyping was not conducted, we could not assign scats to individual weasels. Multiple samples may therefore have originated from the same individuals across repeated transect surveys, so independence among fecal samples cannot be fully verified. Molecular approaches such as DNA metabarcoding now complement traditional methods by improving taxonomic resolution and detectability of soft-bodied prey [34,89,90]. However, these approaches introduce their own interpretive limitations and caveats. Sequence read abundance does not directly represent biomass intake or energetic contribution, and primer bias, reference databases, and contamination remain important constraints [91]. Consequently, using a well-conceived analytical framework is crucial when using feces to infer dietary patterns across seasons and habitats [34,35,89].

Future studies may further elucidate site and seasonal variation in Japanese weasel diet by integrating stable isotope analysis to reflect assimilated diet over a longer time window [92]. Molecular tools, such as fecal DNA genotyping [93,94] could also be used to link scats to the sex or identity of individuals sampled [95].

## 5. Conclusions

Our findings corroborate the emergent view that the Japanese weasel is capable of highly adaptive trophic plasticity, switching its diet to align with locally and seasonally available resources, including plant material [23,52]. This is more similar to the dietary habits of larger *Martes* spp. [66] than to those of other *Mustela* spp. Adult male Japanese weasels average c. 400 g and can reach 800 g [96], with females somewhat lighter, which is considerably heavier than other temperate zone *Mustela* species.

From a conservation and management perspective, our results highlight the importance of maintaining habitat heterogeneity in managed wetlands, where reed beds, levees, paths, and adjacent terrestrial vegetation at WYW enabled Japanese weasels to exploit a diversity of prey types [97]. Such habitat diversity and associated spatial subsidies are also conducive to maximizing site biodiversity [98]. However, management favoring a broader diversity of small carnivore species, especially adaptable invasive species such as the introduced North American raccoon (*Procyon lotor*), masked palm civet (*Paguma larvata*), or the Siberian weasel as a direct competitor, would be disadvantageous to Japanese weasels if they promote intra-guild competition [22].

More broadly, our findings reinforce the evolutionary ecology perspective that dietary niches are emergent properties of consumer–landscape interactions rather than fixed outcomes of morphology or physiology alone [99]. Persistent trophic flexibility illustrates how selection favors behavioral generalism as a strategy for exploiting spatially and temporally heterogeneous environments, allowing species and populations to integrate multiple energy pathways without niche specialization [100]. Such flexibility enhances population persistence and dampens demographic variability under fluctuating resource regimes [101]. Within trait-based and meta-ecosystem frameworks, landscape structure shapes functional diversity, stabilizes food webs, and underpins biodiversity resilience in increasingly human-modified systems [101,102].

## Figures and Tables

**Table 3:** Relationships between occurrences of food categories and principal components PC1 and PC2 based on a correlation matrix across three sites over four seasons. More positive values indicate a greater representation of the item on the corresponding PC axis. Larger absolute loading values represent stronger associations with the seasonal (PC1) or habitat-related (PC2) dietary patterns.

Food Category	Abbreviation	PC1	PC2
Mammals	Mam	−0.372	−0.209
Birds	Brd	−0.370	0.166
Reptiles and amphibians	Herp	0.410	0.021
Fish	Fsh	−0.355	−0.306
Insects	Ins	0.231	0.308
Crustaceans	Cru	0.417	−0.287
Other animals	OthA	0.429	0.052
Seeds (fruits)	Frt	−0.115	0.572
Other plants	OthP	−0.073	0.572

**Table 4:** Results of PERMANOVA with pairwise comparisons among seasons, based on Bray–Curtis dissimilarity calculated from diet composition.

Test	*R* ^2^	*F*	*p* Value
Autumn–Winter	0.170	0.819	0.600
Autumn–Spring	0.082	0.356	0.700
Autumn–Summer	0.074	0.322	0.700
Winter–Spring	0.145	0.678	0.700
Winter–Summer	0.221	1.134	0.300
Spring–Summer	0.179	0.872	0.500

PERMANOVA was conducted with 999 permutations.

**Table 5:** Results of the two-factor PERMANOVA testing the effects of study site and season on diet composition, based on Bray–Curtis dissimilarity.

**Factor**	*df*	*SS*	*R* ^2^	*F*	*p* Value	
Site	2	1.130	0.643	13.023	0.001	**
Season	3	0.366	0.208	2.810	0.038	*
Residual	6	0.260	0.148	—	—	
Total	11	1.757	1.000	—	—	

* *p* ≤ 0.05; ** *p* ≤ 0.01. PERMANOVA was conducted with 999 permutations.

## Data Availability

Data supporting the findings of this study are available from the senior author, Y.K. (ykaneko7946@gmail.com), upon reasonable request.
